# Lead Exposure and Oxidative Stress—A Life Course Approach in U.S. Adults

**DOI:** 10.3390/toxics6030042

**Published:** 2018-08-01

**Authors:** Emmanuel Obeng-Gyasi

**Affiliations:** Department of Built Environment, North Carolina Agricultural and Technical State University, Greensboro, NC 27411, USA; eobenggyasi@ncat.edu; Tel.: +1-336-285-3132

**Keywords:** lead exposure, oxidative stress, life course, lead pollution, lead and health

## Abstract

Lead exposure and a marker of oxidative stress (gamma-glutamyl transferase—GGT), and their effects on life course variables (age, country of birth, education levels, gender, ethnicity, income, and occupation) were explored in this cross-sectional study of United States (U.S.) adults’ ≥ 20 years of age via the National Health and Nutrition Examination Survey (NHANES) 2007–2010 datasets. Country of birth, education levels, gender, ethnicity, income, and occupation showed significant differences depending on the degree of lead exposure, with higher levels of exposure resulting in worse outcomes. Age and GGT were significantly associated with lead exposure. More must be done to mitigate sources of lead exposure, to prevent it from altering the life course of at-risk populations.

## 1. Introduction

The life course theory is one that can be used to understand the factors that affect lead exposure risks and disease outcomes. It can be defined in this context as a study of long-term “biological, behavioral, and psychosocial processes that link adult health and disease risk to physical or social exposures acting during gestation, childhood, adolescence, earlier in adult life or across generations” [1]. The theory states that developmental processes such as delayed development or environmental conditions in utero, which are averse to proper growth and development, are associated with an increased risk of middle and later life illnesses [1,2].

In the model, there is a belief that many of the chronic and adverse health outcomes are created early in development in utero and then bring forth lasting damage to the adult later on. The theory also speaks of damaging physical and social environments which create pathology and induce toxic changes in the body that affects the individual from in utero through adulthood due to cumulative exposure [3]. Modification of the adverse experience is key in mitigating the effects of the exposure [1].

Time and place are key components of the life course framework. Time refers to not only lifetime (i.e., chronological age) but also historical time (i.e., a birth cohort). The birth cohort is the year of birth during which important environmental conditions occur that can affect the health of children in the present and could manifest at a later stage of life [1].

Place is regarding a geographical location and group membership is relating to “family, friends or age, and on the basis of class, ethnicity, residence, and gender that arise out of the social and economic structure of society” [4].

The changing environment one is exposed to determines and affects their risk of disease and how they respond to that risk [1]. Accumulation of risk is a concept of the life course approach which posits that insults build up due to injury, illness, environmental conditions and health behaviors. The life course approach has the objective of testing the extent of cumulative damage to the body as the severity and duration of the exposure increases as the body grows older, it is less likely to handle the damage or repair it [4]. Ultimately, the life course approach offers perspective on disease inclinations, and for “gender, ethnic, and geographical, inequalities in health” [4] and is a means to understand lead exposure among U.S. adults.

Lead, a toxic metal, induces numerous adverse clinical outcomes in children and adults. Due to the widely held view that lead had the capacity to enhance engine performance by boosting octane ratings, reduce engine knocking, and optimize the performance of valve seats within motors, United States motor vehicles used gasoline containing tetraethyl lead additives from the 1920s to 1995 [5]. It plays no role in normal human physiology and through various mechanisms, most involving taking calcium’s place in mechanisms of physiological importance, acts to induce adverse clinical outcomes [6,7]. Additionally, because lead persists in the environment, populations can remain exposed in areas where it was used previously. Lead exposure has decreased significantly over the past 30 years in the U.S. because of policy-driven changes which has caused lead content to be reduced in gasoline, household paint, the food canning process, industrial emissions, and in water [8]. However, despite the dramatic fall in lead exposure in the U.S., certain segments of the population continue to be exposed to elevated levels of lead due to their socioeconomic status, occupation, their place of residence (especially, living in disadvantaged neighborhoods), and/or their history of exposure [8].

Oxidative stress, a process which produces reactive oxygen species (ROS) or reactive nitrogen species (RNS), is an imbalance between the pro-oxidants and antioxidants in the body [9]. Pro-oxidants may be either exogenous or endogenous. The harmful effects of free ROS and RNS are potential biological damage where there is either a disproportionate production of ROS/RNS and/or a deficiency of antioxidants. The redox stress/oxidative stress is a complex process which impacts humans depending on the type of oxidant, on the composition and activities of various antioxidants on the site and intensity of action, and on the ability of repair systems [9].

Under normal conditions, the physiologically important intracellular levels of reactive oxygen species (ROS) are maintained at low levels by various enzyme systems participating in the in vivo redox homeostasis.

Oxidative stress affects the vascular system, a system important to normal physiological function. With respect to vascular oxidative stress, reactive oxygen species (ROS) including such enzymatic processes as xanthine oxidase, Nicotinamide adenine dinucleotide phosphate (NADPH) oxidase, and an uncoupled endothelial nitric oxide (eNOS) synthase, that possibly affects vascular tone or function, by altering it through its effects on nitric oxide (NO) signaling or bioavailability [10]. NO that is released from endothelial cells works in concert with prostacyclin to inhibit platelet aggregation. Specifically, the NO inhibits the attachment of neutrophils to endothelial cells and the expression of adhesion molecules. In elevated concentrations, NO inhibits the multiplying of smooth muscle cells; thus whenever NO deficit is encountered, atherosclerosis may be initiated or potentially accelerated [10]. With respect to the connection between antioxidant nutrients and lead exposure, Hsu and LeonGuo [11] found that lead-induced oxidative stress plays a role in the pathogenesis of lead poisoning by affecting the delicate antioxidant/pro-oxidant equilibrium in cells. They found that in vivo studies suggest lead exposure induces the generation of ROS and alteration of antioxidant defense systems in occupationally exposed workers. Various markers exist for oxidative stress including erythrocytes glutathione (GSH) levels, plasma malondialdehyde (MDA), nitrite/nitrate (NOx) and homocysteine (Hcy) levels, as well as serum ceruloplasmin (Cp), total antioxidants (TAO), endothelin-1 (ET-1) levels and γ-glutamyl transferase (GGT) [12]. In this study, GGT levels will be used as the marker of oxidative stress. Epidemiological studies have consistently suggested that serum GGT within its normal range might be an early and sensitive enzyme related to oxidative stress [13,14].

### Operationalization of Theory

**Figure 1:**
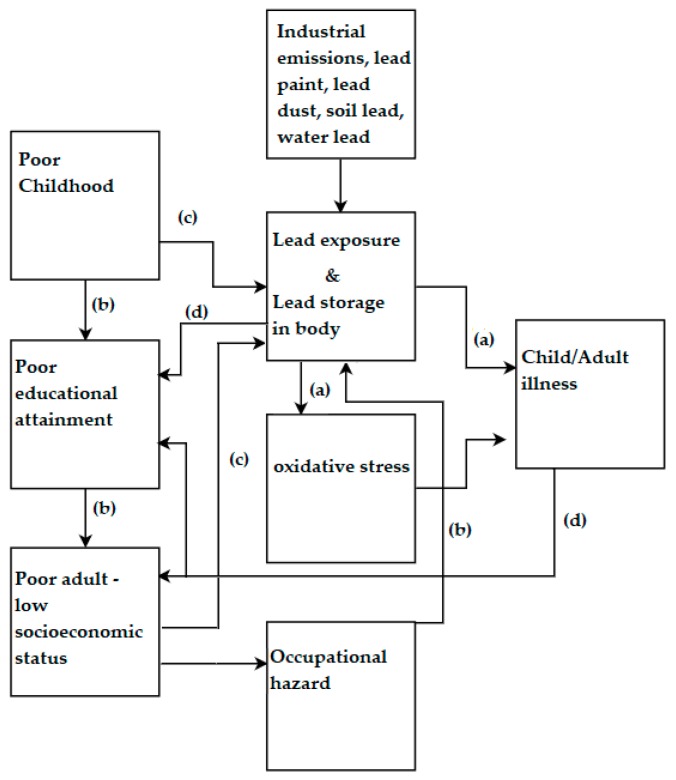
Schematic showing how lead exposure acts across the life course of an individual.

The life course theoretical model will be operationalized in this study as shown in Figure 1 using variables and conditions widely reported in the literature [15,16,17,18].

In the diagram, path A represents a biological pathway where the lead exposure is associated with adverse health outcomes in childhood and adulthood. It should be noted that lead has a half-life of up to 30 years in bone; thus, exposure in utero can continue to accumulate and induce health effects over one’s lifespan. Path B is a social pathway where one’s socioeconomic background increases their risk of exposure to lead which can subsequently accumulate over a lifetime and in the presence of new and acute exposures induce adverse health effects. Path C is the sociobiological path that indicates the social and economic status of an individual and how continuous exposure can increase over a lifetime and subsequently induces disease. Path D represents a biosocial pathway in which the consequences of extensive lead exposure results in decreased educational attainment and subsequently brings about poverty. Ultimately, what this model shows is that exposure during a key period may result in the irreversible induction of pathology [19] which affects an individual through their lifetime. Regarding lead, when it damages some biological functions, especially in the neurological system, the damage may be irreversible making prevention vital.

Prevention starts early, during in utero as toxicity associated with lead crosses the placental barrier and the fact that it competes with calcium, thus affecting fetal and maternal bone function [20]. Maternal bone is an endogenous source of lead; since a woman who has accumulated lead during her lifetime and in her reproductive years, may have a significant store of lead in her bones. This accumulation suggests that women who have been exposed to lead in the past are at risk of exposing the developing fetus to lead via cord blood and after birth, through breast milk [21]. Lead exposure can then start to build up through childhood and into adulthood.

Adult Blood Lead Epidemiology Surveillance (ABLES), which monitors adult blood lead levels (BLL) in the United States [22], adopted 5 μg/dL as the reference for elevated BLLs in adults. Lead exposure among adults mainly occurs in the workplace within lead and zinc ore mining, painting, and battery manufacturing industries. Exposure in these and other industries can occur over the course of one’s life and through mechanisms including oxidative stress and induce pathology. The current study examined lead exposure and sought to determine if it alters key life course variables in a nationally representative sample. It also sought to understand the role of oxidative stress; one of the underlying mechanisms for much of the pathology, due to lead exposure.

## 2. Materials and Methods

Data from NHANES 2007–2010 were used to study lead and oxidative stress biomarker GGT in the general U.S. adult population. The NHANES 2007–2010 survey, conducted by the Centers for Disease Control and Prevention (CDC), used a representative sample of the U.S. noninstitutionalized civilian population. Participants were selected using a complex sampling methodology. The sample weights for NHANES 2007–2010 were based on population estimates that incorporate the national census count. In all, 12,153 adult subjects ≥ 20 years were involved in this complex, multistage, stratified cluster survey in 2007–2010, which after considering sampling weights consisted of 217,057,187 people. Of the 12,153 participants, BLL was measured in 9781 adult subjects which represented an estimated 182,052,299 people.

NHANES 2007–2010 consisted of a standardized questionnaire and a physical examination at a Mobile Examination Center (MEC). The data is available at the organization’s homepage. Methods for data collection are on the NCHS’ website [National Center for Health Statistics, 2007–2008, 2009–2010].

### 2.1. Biomarkers in the Operationalized Theoretical Model

The biomarker of interest is blood lead, which is representative of current lead exposure [23]. Blood lead level (BLL) can help determine the level of exposure but is not representative of the body burden of lead. Other biomarkers include GGT, which can be used as a measure of oxidative stress. In NHANES 2007–2010, the biochemistry biomarkers were measured via the Roche Modular P chemistry analyzer at the University of Minnesota, MN, USA and a Beckman Synchron LX20, Beckman UniCel^®^ DxC800 Synchron (Brea, CA, USA) at Collaborative Laboratory Services. Metal assays in whole blood samples were performed in the NHANES 2007–2010 at the Division of Laboratory Sciences, National Center for Environmental Health (NCEH) of the CDC.

### 2.2. Statistical Analysis

In this study, the analysis was performed on those experiencing various degrees of exposure represented by BLLs in three tertiles; 0–2 µg/dL, 2–5 µg/dL, 5–10 µg/dL, presented as tertile 1, tertile 2, and tertile 3 in this study.

The association between lead and the oxidative stress marker were examined with linear regression. Natural log transformation was used for independent and dependent variables in regression analysis as the variables of interest were not normally distributed.

Data analysis and management was done in accordance with the NHANES analytical guidelines relating to its survey design and weighting. The software Stata SE/15.0 (StataCorp, College Station, TX, USA) was used.

A *p*-value of <0.05 was considered significant while a value of <0.10 was considered moderately significant.

## 3. Results

### 3.1. Country of Birth

Results for the country of birth over the tertiles of exposure are shown in Table 1. Those born in Mexico were disproportionately represented among those in the higher tertiles of exposure.

### 3.2. Education Level

The educational level categories of the surveyed participants included those who attained less than Grade 9, those with 9th to 11th Grade educational level (including 12th grade), those with a High School diploma, those with some College or Associate Degree (AA), and those who completed College. The complete results of the surveyed participants’ educational attainment are displayed in Table 2 below.

### 3.3. Gender

The results of the different gender categories and tertiles of exposure are shown (in percentages) in Table 3 below.

### 3.4. Ethnicity

The ethnic groups found in the data included Mexican American, Other Hispanic, Non-Hispanic White, Non-Hispanic Black, and Other Race which included multi-racial. The results are shown (in percentages) in Table 4 below.

### 3.5. Income

Income and its role in lead exposure is demonstrated in Table 5. Trends show that those of lower incomes were represented in higher proportions in the lowest income groups.

### 3.6. Occupation

Occupation and its manifestation in the teritles of exposure are shown in Table 6. Note that agriculture, forestry and fishing, along with construction had a higher representation in the highest tertiles of exposure.

### 3.7. Age and Oxidative Stress

Age and oxidative stress biomarker GGT and their relation with BLL over the tertiles of exposure are presented in Table 7 below.

The associations of BLL, presented as the natural log of BLL (lnBPb) with age and GGT are presented in Table 8 below.

## 4. Discussion

### 4.1. Lead Oxidative Stress and the Life Course

Lead exposure can be understood through a life course approach. Exposure can begin in utero with risk continuing into adulthood. The 30-year half-life of lead in bone, an endogenous source of lead exposure, is such that exposed populations remain exposed during their lifetime. The current study, one of the first to examine lead exposure, oxidative stress, and life course variables in a nationally representative sample had several key findings. Regarding country of birth, those born in Mexico had significantly higher lead exposure than those born in the U.S. One may refer to the sociobiological pathway where being born in Mexico, which has not enjoyed the regulatory successes [24] of the U.S., may leave some populations exposed to excess levels of lead. In addition, Mexican-Americans and Non-Hispanic Blacks were represented in higher proportions in the higher exposure tertiles than others. Those with better education also had less lead exposure. The work of Bellinger and colleagues [25] which found that slightly elevated blood lead levels around the age of 24-months were associated with intellectual and academic performance deficits at age 10-years. In this study, those of lower income were represented in more in the highest tertile of exposure. One may refer to the biosocial pathway of the life course approach where potentially altered biology lowers one’s socioeconomic status. It also speaks to the social pathway where one’s socioeconomic status increases their risk of exposure. Agriculture, forestry and fishing along with construction were more represented in the highest tertile of exposure as compared to other occupations. The construction industry has historically been a source of lead exposure among adults [26]. Regarding gender, males were more represented in higher tertiles of lead exposure than lower tertiles. Accumulation of risk was demonstrated in this study as higher doses of exposure resulted in more adverse outcomes for sociodemographic and clinical indicators.

The current study found a statistically significant association between lead exposure and oxidative stress bio-marker GGT. The research of Lee and co-authors backs this as they found associations, in an analysis of NHANES III, between blood lead and GGT in adults [27]. One of the consequences of lead exposure is oxidative stress. Generation of ROS/RNS via the mitochondrion [28] has potentially severe consequences. The consequences of oxidative stress in the life course is such that it can contribute to the pathogenesis of various diseases such as neurodegenerative diseases [29], cardiovascular diseases [30,31], hepatobiliary diseases [32], and renal disease [33]. The ROS/RNS produced in the tissues can inflict direct damage to macromolecules, such as lipids, nucleic acids, and proteins [34] which brings forth much of the pathology associated with oxidative stress.

### 4.2. Limitations

Measurement of BLLs does not indicate longer-term exposure; rather, it is indicative of recent lead exposure as well as lead that has been mobilized from bone or other tissue sources with no ability to distinguish between both. Measuring of bone lead levels, particularly tibia lead level, via K-Shell X-Ray Fluorescence (KSXF) would have provided more information on the length of exposure as bone lead levels are indicative of long-term cumulative exposure to lead. Both the BLLs and bone lead levels, taken together, would have provided the best and most comprehensive view of the participant’s exposure. [35] The current cross-sectional study depicts only a snapshot in time. A longitudinal study of the group may have yielded different results as people’s unique circumstances (finances, family, etc.) change resulting in people moving to different parts of the country, creating new social networks, gaining access to necessary knowledge about lead exposure and hence relocating to safer areas in which exposure is limited.

## 5. Conclusions

The life course approach may be explained lead exposure during the one’s lifetime. Exposure to lead increases with age; as does oxidative stress. The current study found a significant association between lead exposure and oxidative stress and age. The trajectory of one’s life may be significantly altered, starting from where they were born, which may result in the exposure to toxicants such as lead which could subsequently affect biological systems, for example, the neurological, cardiovascular, and hepatic system, ultimately altering income, education levels, and occupation. In U.S. adults, this data suggests that such changes may affect ethnic minorities more so than others. More must be done to mitigate lead exposure for vulnerable populations in order to promote the human development of all in, line with national development goals.

## Figures and Tables

**Table 1:** Country of Birth and Tertiles of Exposure.

Exposure Level	Born in 50 US States (±SE%)	Born in Mexico (±SE%)	Born in Other Spanish Speaking Country (±SE%)	Born in Other Non-Spanish Speaking Country (±SE%)
*N*	9993	621	480	1054
Tertile 1	76.9% (1.1)	63.7% (2.2) +	79.2% (2.7)	66.7% (2.8)
Tertile 2	20.8% (1.0)	29.9% (1.7) ++	18.9% (2.7)	30.1% (2.4)
Tertile 3	2.1% (0.2)	5.2% (0.9) *	1.5% (0.3)	2.6% (0.6)
Total	100%	100%	100%	100%

+ *p* < 0.05 significant difference between those born in Mexico as compared to those born in the U.S. and Other Spanish speaking countries. ++ *p* < 0.05 significant difference between those born in Mexico as compared to those born in the U.S. or other Spanish speaking countries. * *p* < 0.05 significant difference between those born in Mexico and those born U.S., other Spanish speaking countries, and other non-Spanish speaking countries.

**Table 2:** Education Level and Tertiles of Exposure.

Exposure Level	Less Than 9th Grade (±SE%)	9–11th Grade (Includes 12th Grade) (±SE%)	High School Grad/GED Equivalent (±SE%)	Some College or AA Degree (±SE%)	College Graduate or Above (±SE%)
*N*	802	1600	2928	3587	3215
Tertile 1	61.1% (2.3) *	67.4% (2.2) +	73.8% (1.2) ^	79.1% (1.0) ^^	80.3% (1.4) ^^^
Tertile 2	32.1% (2.0) **	28.6% (2.1) ++	23.9% (1.2)	18.5% (0.9)	18.4% (1.3)
Tertile 3	5.9% (0.8) ***	3.1% (0.4) +++	2.0% (0.4)	2.3% (0.4)	1.1% (0.3)
Total	100%	100%	100%	100%	100%

* *p* < 0.05 significant difference between less than 9th grade and all other education categories. ** *p* < 0.05 significant difference between less than 9th grade and High school graduate/GED equivalent, Some college or AA degree, and college graduate or above. *** *p* < 0.05 significant difference between less than 9th grade and all other educational attainments in exposure group. + *p* < 0.10 moderately significant difference between 9th and 11th grade (including 12th grade) and High school graduate/GED equivalent. ++ *p* < 0.05 significant difference between 9th and 11th grade (including 12th grade), some college or AA degree, and college graduate or above. +++ *p* < 0.05 significant difference between 9th and 11th grade (includes 12th grade) and High School graduate/GED equivalent, and College graduate or above. ^ *p* < 0.05 significant difference between High school graduate/GED equivalent and all other educational categories. ^^ *p* < 0.05 significant difference between some college or AA degree and High School graduate/GED equivalent, 9–11th grade (including 12th grade), and Less than 9th grade. ^^^ *p* < 0.05 significant difference between college graduate or above and high school graduate/GED equivalent, 9–11th grade (includes 12th grade), and less than 9th grade.

**Table 3:** Gender and Tertiles of Exposure.

Exposure Level	Male (±SE%)	Female (±SE%)
*N*	5858	6295
Tertile 1	67.8% (1.2)	82.5% (0.9) *
Tertile 2	27.8% (1.0) **	16.6% (0.8)
Tertile 3	3.7% (0.3) ***	0.9% (0.1)
Total	100%	100%

* *p* < 0.05 Female representing a significantly larger proportion than Male in this exposure group. ** *p* < 0.05 Male representing a significantly larger proportion than Female in this exposure group. *** *p* < 0.05 Male representing a significantly larger proportion than Female in this exposure group.

**Table 4:** Ethnicity and Tertiles of Exposure.

Exposure Level	Mexican American (±SE%)	Other Hispanic (±SE%)	Non-Hispanic White (±SE%)	Non-Hispanic Black (±SE%)	Other Race-Including Multi-Racial (±SE%)
*N*	1032	600	8342	1377	802
Tertile 1	72.0% (1.9)	80.7% (2.1) *	76.9% (1.2) **	71.5% (1.3)	66.7% (3.4)
Tertile 2	23.2% (1.4) ***	17.3% (2.4)	21.1% (1.1)	24.4% (1.2) +	29.7% (2.9) ****
Tertile 3	3.7% (0.7) ++	1.6% (0.3)	1.8% (0.2)	3.7% (0.5) +++	2.9% (0.8)
Total	100%	100%	100%	100%	100%

* *p* < 0.05 significant difference between Other Hispanic and Mexican-American, Other Hispanic and non-Hispanic Black and Other Hispanic and Other Race-including multi-racial. ** *p* < 0.05 significant difference between non-Hispanic White and Mexican-American, non-Hispanic White and Non-Hispanic Black and non-Hispanic White and Other Race-Including Multiracial. *** *p* < 0.05 significant difference between Mexican-American and Other Hispanic. **** *p* < 0.05 significant difference between other race-including multiracial and Mexican American, Other Hispanic, and non-Hispanic white. + *p* < 0.05 significant difference between Non-Hispanic Black and Other Hispanic and non-Hispanic White. ++ *p* < 0.05 significant difference between Mexican American and other Hispanic and non-Hispanic white. +++ *p* < 0.05 significant difference between non-Hispanic Black and Other Hispanic, non-Hispanic White.

**Table 5:** Income and Tertiles of Exposure.

Income	*N*	Tertile 1 (±SE%)	Tertile 2 (±SE%)	Tertile 3 (±SE%)
$0 to $4999 ^	273	2.2% (0.3)	2.8% (0.5)	4.0% (1.2)
$5000 to $9999 ^	399	3.2% (0.2)	4.8% (0.4)	5.7% (1.5)
$10,000 to $14,999 ^	689	5.5% (0.5)	7.7% (0.8)	13.1% (2.4)
$15,000 to $19,999 ^	649	5.2% (0.5)	7.9% (0.7)	6.9% (1.7)
$20,000 to $24,999 +	799	7.3% (0.4)	7.9% (0.7)	5.1% (1.5)
$25,000 to $34,999 +	1212	10.5% (0.6)	12.5% (0.8)	11.6% (1.9)
$35,000 to $44,999 +	1032	9.4% (0.6)	9.6% (0.6)	10.1% (2.0)
$45,000 to $54,999 +	900	8.5% (0.7)	8.1% (0.8)	6.5% (2.2)
$55,000 to $64,999 +	782	7.3% (0.4)	6.9% (0.9)	9.2% (2.7)
$65,000 to $74,999 +	670	6.7% (0.5)	4.6% (0.7)	5.8% (2.2)
$75,000 to $99,999 +	1306	12.8% (0.8)	9.6% (1.0)	8.1% (2.3)
$100,000 and Over +	2256	21.4% (1.3)	17.6% (1.5)	14.0% (3.8)
Total	10,967	100%	100%	100%

^ Increasing percentage trend between lower and higher exposure groups. + decreasing percentage trend between lower and higher exposure groups.

**Table 6:** Longest Held Occupation and Teritles of Exposure.

Occupation	Tertile 1 (±SE%)	Tertile 2 (±SE%)	Tertile 3 (±SE%)
Accommodation, Food Services	80.8% (2.7)	17.7% (2.7)	1.4% (0.5)
Agriculture, Forestry, Fishing	53.2% (3.5)	41.8% (3.9) *	4.8% (2.3) **
Construction	58.2% (3.0)	35.4% (2.7) *	5.1% (1.1) **
Finance, Insurance	78.0% (3.4)	19.9% (3.4)	1.8% (1.6)
Information	71.7% (4.3)	27.6% (4.2)	0.2% (0.2)
Manufacturing: Durable Goods	66.3% (2.3)	28.6% (2.1)	4.4% (0.7)
Manufacturing: Non-Durable Goods	68.6% (2.6)	28.4% (2.4)	2.8% (0.8)
Public Administration	68.8% (3.2)	29.4% (3.1)	1.0% (0.6)
Real Estate, Rental, Leasing	83.0% (5.4) +	13.8% (4.8)	2.7% (1.8)
Retail Trade	79.1% (2.5)	18.3% (2.2)	2.0% (0.7)

* *p* < 0.05 of Agriculture, Forestry, Fishing and Construction making up a significantly larger proportion of exposure category as compared to other industries in exposure category. ** *p* < 0.05 for Agriculture, Forestry, Fishing, and construction making up a significantly larger proportion of exposure category as compared to many other industries in exposure category. + *p* < 0.05 Real Estate, Rental, Leasing making up a significantly larger proportion of exposure category as compared to many other industries in this category.

**Table 7:** Age and Clinical Factors and Tertiles of Exposure.

Variables	Tertile 1 (±SE)	Tertile 2 (±SE)	Tertile 3 (±SE)
BLL	1.09 (0.01)	2.78 (0.02)	6.40 (0.10)
Age *	44.25 (0.32)	56.05 (0.54)	54.77 (1.13)
GGT **	26.54 (0.40)	33.10 (0.92)	40.70 (3.77)

* Significant difference between tertile 1 and 2, 3. ** *p* < 0.05 significant difference between Tertile 1 and 2, 3.

**Table 8:** Age and Clinical Factors and Tertiles of Exposure.

Variables	lnBPb Adjusted (95% CI)	*p*-Value
GGT +	0.050 (0.015, 0.085)	0.007
Age *	0.827 (0.761, 0.893)	0.0001

+ Adjusted for age, ethnicity, gender, country of birth, education, income, alcohol consumption, smoking, and BMI. * Adjusted for ethnicity, gender, country of birth, education, income, alcohol consumption, smoking, and BMI.
